# Integrated genome-based assessment of safety and probiotic characteristics of *Lactiplantibacillus plantarum* PMO 08 isolated from kimchi

**DOI:** 10.1371/journal.pone.0273986

**Published:** 2022-10-03

**Authors:** Young Joo Oh, Seul-Ah Kim, Soo Hwi Yang, Da Hye Kim, Ya-Yun Cheng, Jung Il Kang, Sang Yun Lee, Nam Soo Han

**Affiliations:** 1 Pulmuone Co., Ltd., Cheongju, Republic of Korea; 2 Brain Korea 21 Center for Bio-Health Industry, Division of Animal, Horticultural, and Food Sciences, Chungbuk National University, Cheongju, Chungbuk, Republic of Korea; Universidad Autonoma de Chihuahua, MEXICO

## Abstract

*Lactiplantibacillus plantarum* PMO 08 has been used as a probiotic starter culture for plant-based fermented beverages, with various health-promoting effects such as cholesterol-lowering and anti-inflammatory activities. This study aimed to analyze the genome sequence of *Lp*. *plantarum* PMO 08 and identify its safety and probiotic characteristics at the genomic level. For this, complete genome sequencing was conducted to investigate the genes associated with risk and probiotic characteristics by using Pacbio combined with Illumina HiSeq. This bacterial strain has one circular chromosome of 3,247,789 bp with 44.5% G + C content and two plasmids of 50,296 bp with 39.0% G + C content and 19,592 bp with 40.5% G + C content. Orthologous average nucleotide identity analysis showed that PMO 08 belongs to the *Lp*. *plantarum* group with 99.14% similarity to *Lp*. *plantarum* WCFS1. No deleterious genes were determined in the virulence factor analysis, and no hemolysin activity or secondary bile salt synthesis were detected *in vitro* test. In the case of antibiotic resistance analysis, PMO 08 was resistant to ampicillin *in vitro* test, but these genes were not transferable. In addition, the strain showed same carbohydrate utilization with *Lp*. *plantarum* WCFS1, except for mannopyranoside, which only our strain can metabolize. The strain also harbors a gene for inositol monophosphatase family protein related with phytate hydrolysis and have several genes for metabolizing various carbohydrate which were rich in plant environment. Furthermore, in probiotic characteristics several genes involved in phenotypes such as acid/bile tolerance, adhesion ability, and oxidative stress response were detected in genome analysis. This study demonstrates that *Lp*. *plantarum* PMO 08 harbors several probiotic-related genes (with no deleterious genes) and is a suitable probiotic starter for plant-based fermentation.

## 1. Introduction

Probiotics are live organisms, often bacteria, with beneficial health effects such as lowering cholesterol and modulating of gut-microbiota when consumed in sufficient amounts [1]. In the last few decades, many probiotics have been developed from dairy products and used as starter cultures in the fermentation industry. Probiotics from plant-based foods are attracting attention as alternatives due to the increasing limitations of milk-based foods as a result of lactose intolerance and milk allergy for instance [2, 3]. Meanwhile, plant-based products contain anti-nutritional factor, such as phytates, which interfere with mineral absorption [4]. However, fermentation can improve nutritional properties by degrading phytates to improve mineral absorption [5]. For these reasons, scientists have focused on identifying novel food-grade strains with functional properties for plant-based fermentation [6].

*Lactiplantibacillus plantarum* is found in various environmental niches including the gastrointestinal and vaginal tracts, plants, and many food products [7]. This bacterial strain is generally recognized as safe (GRAS) and has been consumed as a fermented food with documented safety, offering diverse applications [8]. Owing to their high tolerance to gastrointestinal conditions and health-promoting activities, many *Lp*. *plantarum* species have been developed as probiotics [9]. Similarly, *Lp*. *plantarum* PMO 08 has been developed as a probiotic starter for plant-based fermented beverages [10]. This strain was isolated from kimchi, a Korean fermented vegetable, with high stability under gastrointestinal conditions [11]. In addition, several studies reported its health-promoting activities such as anti-inflammatory *in vitro* [12], cholesterol-lowering in mice [13], and gut microbiome shaping in human [14]. As many bioinformatics tools have been developed over the past decades, a genetic information of certain bacteria can accelerate probiotic research at the genomic level [15, 16]. Genome analysis can be used for safety assessments, such as the determination of genes for antimicrobial resistance and virulence factors [17]. In addition, probiotic features, such as tolerance to acid and bile salts, adhesion ability to epithelial cells, and antioxidant capacity, can be determined at the genomic level [18]. Furthermore, the availability of complete genome sequences has significantly expanded our understanding of the biology of these microorganisms [19].

The goal of this study was to analyze the whole genome sequence and investigate the safety and probiotic traits of *Lp*. *plantarum* PMO 08 at the genomic level. To achieve this, we conducted whole-genome sequencing of this strain using Pacbio combined with Illumina HiSeq. In addition, we analyzed its COG functional categories, average nucleotide identity (ANI)-based phylogenic taxonomy, and the presence of safety-related genes that encode virulence factors and toxins. Furthermore, we demonstrated the biochemical and probiotic properties of the PMO 08 strain in genomic levels and compared with results obtained from various *in vitro* studies.

## 2. Materials and methods

### 2.1. Microorganism and culture conditions

*Lactiplantibacillus plantarum* PMO 08 was isolated from kimchi [11] and *Lp*. *plantarum* WCFS1 was obtained from American Type Culture Collection (ATCC). The genomic DNA of *Lp*. *plantarum* PMO 08 was extracted from overnight culture in MRS medium (Difco, Detroit, MI, USA) at 37°C using a bacterial genomic DNA prep kit (Solgent, Daejeon, Korea) according to the manufacturer’s instructions. The prepared genomic DNA was kept at -20°C for future use. The quality and quantity of the isolated DNA were determined by SPECTRA MAX 190 (Molecular Devices, CA, USA).

### 2.2. Whole-genome sequencing and genome assembly

Genome sequencing based on hybrid technologies was conducted at Macrogen (Seoul, Korea). A PacBio RS II sequencer (Pacific Biosciences, Menlo Park, CA, USA) was used for long-read sequencing to construct the contigs, and a HiSeq 2500 instrument (Illumina, San Diego, CA, USA) was used for short reads to improve the accuracy of the contigs. Sequencing libraries were generated with a SMRTbell template prep kit v3.0 (Pacific Biosciences) for the PacBio RS II and a TruSeq Nano DNA kit (Illumina) for the HiSeq 2500 following the manufacturer’s protocols. The quality of the raw data sequences was checked using FastQC v0.11.9 [20]. The generated reads were assembled *de novo* using Hierarchical Genome Assembly Process (HGAP v3.0), with a default minimum length of 6 kb [21]. When both ends of the contig overlapped, the contig was regarded as circular, and overlapped regions were manually trimmed. To make the contigs more accurate, a final polishing step was performed using Pilon v1.21 [22]. The completeness of the final assemblies was evaluated using the Benchmarking Universal Single-Copy Orthologs (BUSCO v3.02) software with parameters set to default [23]. Finally, the genome sequence of *Lp*. *plantarum* PMO 08 was annotated using the NCBI Prokaryotic Genome Annotation Pipeline (PGAP v4.11) [24]. All software were run with default settings unless otherwise specified. Further COG functional categories were analyzed using the Eggnog-mapper (v5.0) [25].

### 2.3. ANI-based taxonomy analysis

Species designation of the strain was first determined using 16S rRNA gene using the Ribosomal Database Project Classifier (http://rdp.cme.msu.edu/). Further analysis, Orthologous (Ortho) ANI was calculated using CJ Bioscience’s OrthoANI Tool (OAT), available on the EzBioCloud server (https://www.ezbiocloud.net/tools/orthoani) [26].

### 2.4. Determination of virulence factors and undesirable genes

The presence of genes encoding virulence factors and toxins in the PMO 08 genome was determined using the virulence factor database (VFDB) available at http://www.mgc.ac.cn/cgi-bin/VFs/v5/main.cgi [27]. Two search criteria that is a stringent search using the cut-off values at >80% identity and >60% coverage and a less stringent search with cut-off values >60% similarity, >60% coverage, and E-values <1e-10 were used to identify the possible virulence genes. In addition, the BlastKoala search tool in the Kyoto Encyclopedia of Genes and Genomes (KEGG) database (Release 90.1) available at https://www.kegg.jp/ was used to identify virulence factors and undesirable genes [28].

### 2.5. Determination of antibiotic resistance

The antibiotic resistance of PMO 08 was investigated using both phenotypic and genotypic methods. The resistance phenotype of the strain was tested as recommended by the European Food Safety Authority (EFSA) [29]. The susceptibility of the strain to seven antimicrobial drugs (ampicillin, gentamicin, kanamycin, erythromycin, clindamycin, tetracycline, and chloramphenicol) was determined. The minimum inhibitory concentration (MIC) for each antimicrobial was evaluated using the microdilution method as described in the international standard ISO 10932:201020. Briefly, the strain was grown on MRS agar plates containing each antibiotic for 16–24 h. The antimicrobial solutions were prepared by dissolving each antimicrobial powder in an appropriate solvent and adjusting its potency, as suggested in the ISO standard. *Lacticaseibacillus rhamnosus* GG was used as a quality control strain to ensure the performance of the prepared antimicrobial solutions. The MIC of each antimicrobial was determined in triplicate after incubation for 48 h.

Antibiotic resistance genes were searched using the publicly available databases such as BlastKOALA search tool under “Brite ko01504: Antimicrobial resistance genes” [28], the Comprehensive Antibiotic Resistance Database (CARD) [30], and ResFinder 4.0 [31]. The transferability of the antibiotic resistance genes found in the genome was investigated by their locations in two mobile elements: plasmids and bacteriophages. The existence of prophages in the genome was determined using the PHASTER tool available at http://phaster.ca/ [32] and mobile elements were investigated by using Mobile Element Finder available at https://cge.cbs.dtu.dk/services/MobileElementFinder/ [33].

### 2.6. Biochemical profiles

Carbohydrate utilization patterns were analyzed using the API CHL kit (BioMériux Co., Marcy-l’Étoile, France), according to the manufacturer’s instructions. Briefly, bacterial cells were harvested after cultivation and resuspended in the API 50CH medium. A 120 μL aliquot of the bacterial resuspension was pipetted into each tube of the test strips, and then mineral oil was dropped in the cupules to cover the tubes, followed by incubation at 37°C for 48 h. Fermentation patterns were recorded as positive if the blue indicator in the medium changed to yellow (with the exception of tube #25, which changed to black).

### 2.7. Probiotic-related genes

The sequence information for different probiotic genes [34] of related strains was obtained from the NCBI database and used to identify the probiotic genes present in *Lp*. *plantarum* strains by a sequence similarity search using BLASTP and sequence similarity dropped below 70%.

## 3. Results and discussion

### 3.1. Genomic features

**Fig 1:**
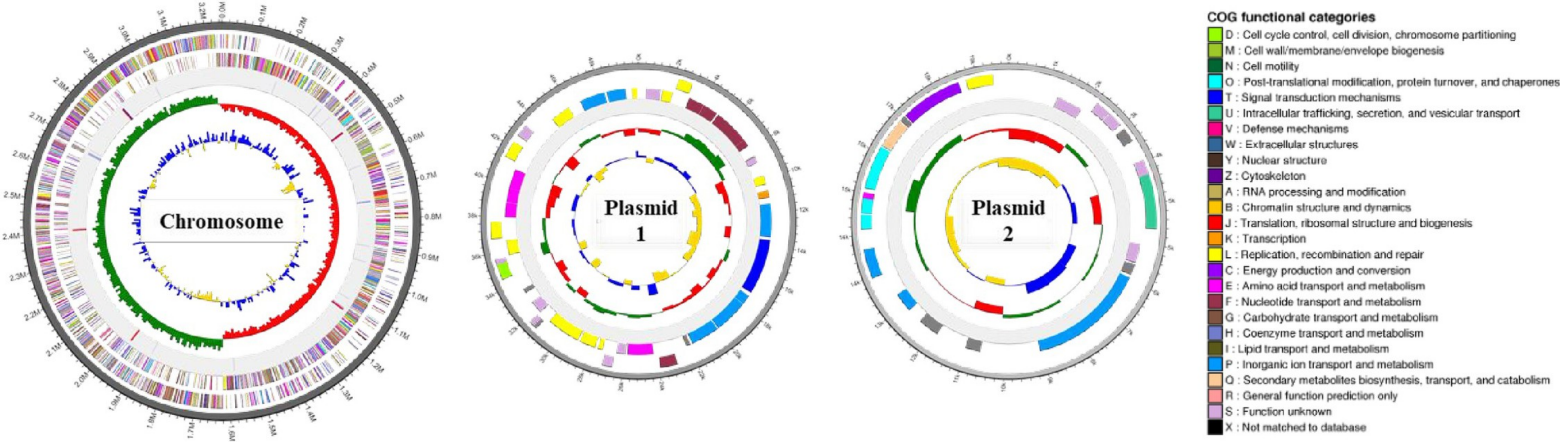
Graphical genome maps of the *Lactiplantibacillus plantarum* PMO 08 chromosome and plasmids. Circles represent the following characteristics from the outermost circle to the center: (1) contig information, (2) coding sequences on forward strand, (3) coding sequences on reverse strand, (4) tRNAs and rRNAs, (5) GC skew, and (6) GC ratio. G, guanine; C, cytosine.

To analyze the genetic characteristics of *Lp*. *plantarum* PMO 08, genomic DNA was sequenced. In PacBio RS II sequencer, total of 152,212 high-quality reads and 1,601,406,015 bp were produced, with an average read length of 10,520 bp. In case of Illumina Hiseq sequencer, total of 8,257,502 reads and 834,007,702 bp were generated. The assembly process generated one circular chromosome of 3,247,789 bp with 44.51% G + C content as well as two plasmids of 50,296 bp with 39.05% G + C content and 19,592 bp with 40.49% G + C content (Fig 1). In addition, genome analysis revealed that *Lp*. *plantarum* PMO 08 contained 2,972 protein-coding genes, 71 tRNA genes, 16 rRNA genes, and four ncRNA genes (Table 1). Notably, no CRISPR sequences were detected. Further COG functional categories are presented in S1 Fig.

**Table 1:** Genomic features of *Lactiplantibacillus plantarum* PMO 08.

Attribute	Total		Chromosome		Plasmid1		Plasmid2	
	Value	% of total	Value	% of total	Value	% of total	Value	% of total
Genome size (bp)	3,317,677	100	3,247,789	97.89	50,296	1.52	19,592	0.59
DNA G+C (bp)	1,473,246	100	1,445,674	98.13	19,640	1.33	7,932	0.54
Total genes	3,121	100	3,055	97.89	42	1.35	24	0.77
CDS (total)	3,030	100	2,964	97.82	42	1.39	24	0.79
CDS (coding)	2,972	100	2,909	97.88	40	1.35	23	0.77
tRNA genes	71	100	71	100.00	0	0.00	0	0.00
rRNA	16	100	16	100.00	0	0.00	0	0.00
ncRNA	4	100	4	100.00	0	0.00	0	0.00
Pseudo genes	58	100	55	94.83	2	3.45	1	1.72
CRISPR sequence	0	0	0	0.00	0	0.00	0	0.00

CDS, coding sequence; ncRNA, non-coding RNA; pseudogenes, nonfunctional segments of DNA; CRISPR sequence, clustered regularly interspaced short palindromic repeat sequences

### 3.2. ANI-based taxonomic analysis

**Fig 2:**
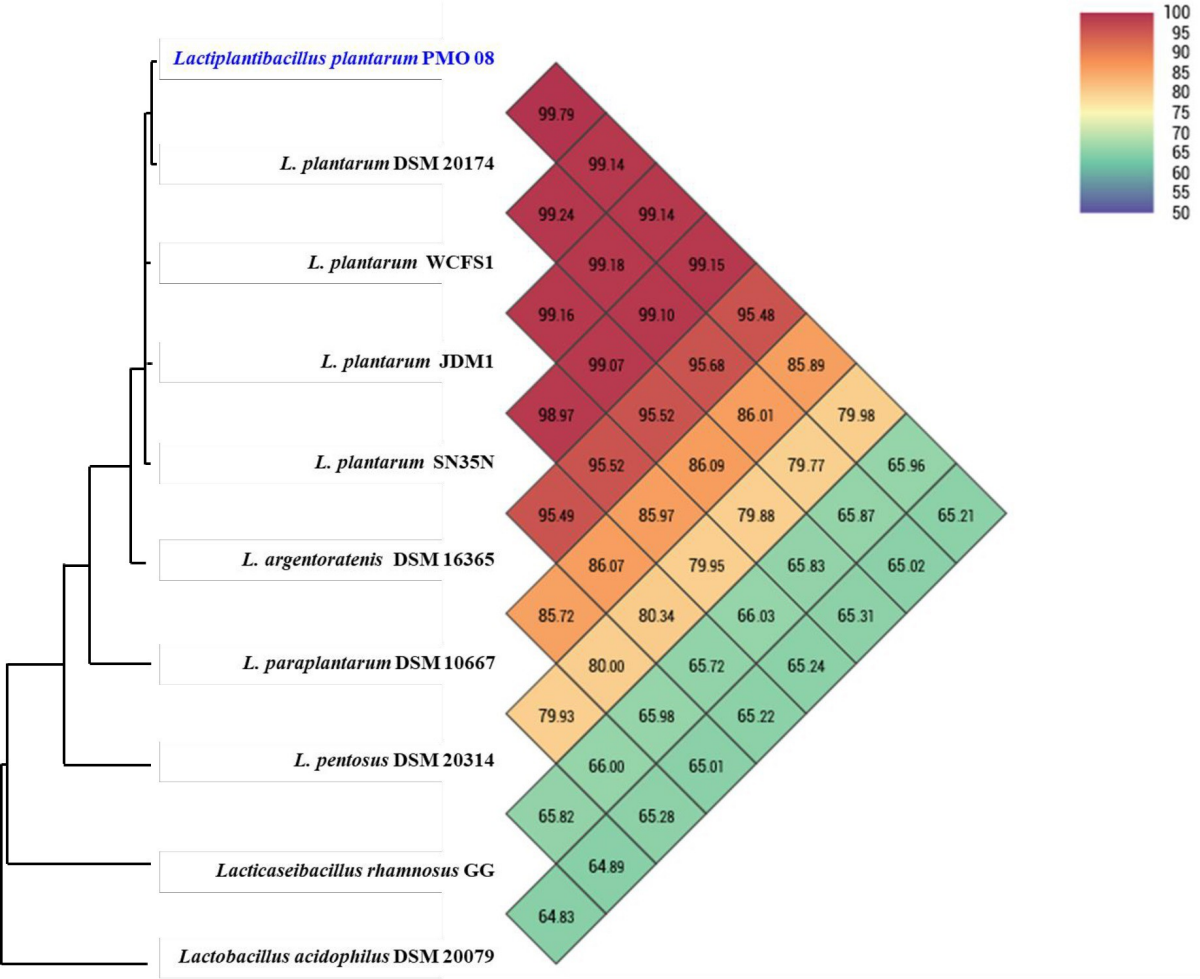
Heat map of OrthoANI of *Lactiplantibacillus plantarum* PMO 08 compared with other lactic acid bacteria. Each cell represents the OrthoANI values between the row and the corresponding genomes of the column. The phylogenetic tree on the left represents a phylogenetic distance constructed by CJ Bioscience’s OAT, available on the EzBioCloud server.

ANI is a measure of nucleotide-level genomic similarity between the coding regions of two genomes, and OrthoANI is an improved version that compares orthologous fragment pairs [26]. The taxonomy of the PMO 08 strain was analyzed by comparing its OrthoANI value with that of other lactic acid bacteria (Fig 2). All *Lp*. *plantarum* strains showed more than 99% OrthoANI values with PMO 08, with the type strain DSM 20174 showing the highest value (99.79%). In general, two genomes are considered the same species when the ANI value is higher than 95–96% [35]. With this, PMO 08 showed 99.14% OrthoANI values with WCFS1 and 65.96% with LGG, which are commercial probiotics. These results indicate that strain PMO 08 belongs to the species *Lp*. *plantarum*. Meanwhile, *Lp*. *argentoratensis* which showed 95.48% OrthoANI with *Lp*. *plantarum* PMO 08 was belonging to *Lactobacillus plantarum* subsp. *argentoratensis*, but recently reclassified as *Lp*. *argentoratensis* sp. nov. [36].

### 3.3 Determination of virulence factor and toxin-encoding genes

The virulence factor database (VFDB) and BlastKoala were used to detect genes that encode virulence factors and toxins that may exist in the PMO 08 genome. No virulence genes were detected under stringent criteria of >80% identity and >60% coverage in VFDB. However, three categories related to virulence factors and undesirable metabolites were detected in BlastKoala (Table 2).

**Table 2:** List of virulence and undesirable genes detected in the genome of *Lactiplantibacillus plantarum* PMO 08.

Metabolism	Product	KEGG ID	Gene	Gene ID	Name
Signaling and cellular processes	Bacterial toxins	K11068	*hlyIII*	QOF00726.1	Type II toxins: membrane damaging toxins
Carbohydrate metabolism (for D-lactate formation)	D-lactate	K03778	*ldhA*	QOF01570.1	D-Lactate dehydrogenase [EC:1.1.1.28]
		K22373	*larA*	QOF03159.1	lactate racemase [EC:5.1.2.1]
Lipid metabolism (primary and secondary bile acid biosynthesis)	Secondary bile acid	K01442	*cbh*	QOF03186.1	Choloylglycine hydrolase [EC:3.5.1.24]
				QOF00553.1	
				QOF00673.1	
Amino acid metabolism (for biogenic amine formation)	ND^1^		*-*	-	-

^1^ND, not detected.

**Fig 3:**
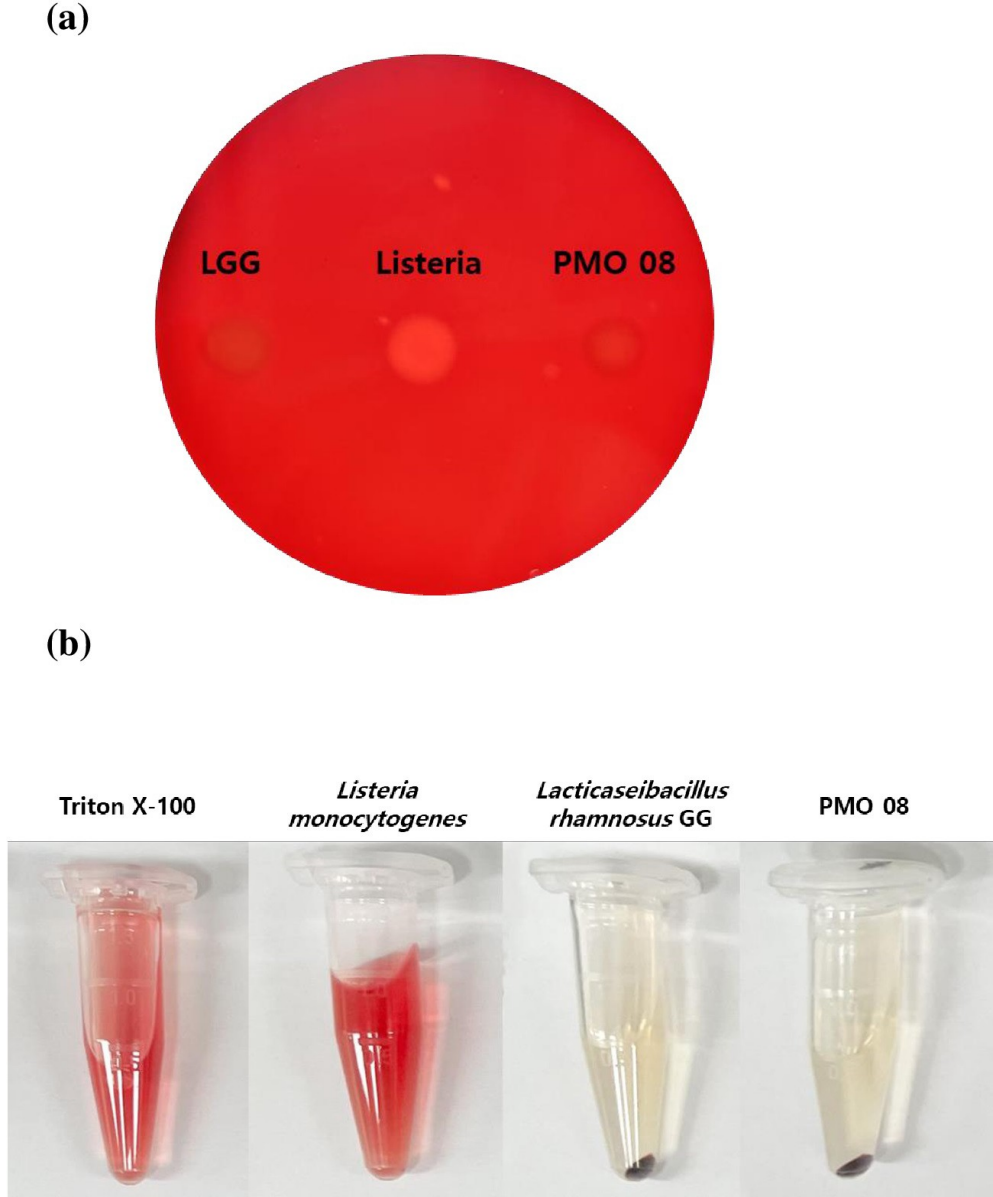
Hemolysin activity of *Lactiplantibacillus plantarum* PMO 08 on erythrocytes. (a) Hemolysin activity of *Lp*. *plantarum* PMO 08 analyzed on a 5% sheep blood agar plate. (b) Hemolysin activity of culture supernatant from *Lp*. *plantarum* PMO 08 treated with erythrocytes. Triton X-100 and *Listeria monocytogenes* used as a positive control and *Lp*. *rhamnosus* GG used as a negative control.

The first category is the bacterial toxin, hemolysin III (*hly*III), which is classified as a membrane-damaging toxin. When the hemolysin activity of PMO 08 was tested using the supernatant and agar plate method, the strain did not show any erythrocyte lysis (Fig 3). In addition, the sequence of *hly*III in PMO 08 was compared with other lactic acid bacteria such as *Lp*. *plantarum* WCFS1, JDM1, and 299v, which are commercial probiotics known to have non-hemolytic activity. As result, *hly*III in PMO 08 showed 100% similarity with the genes of these strains. Furthermore, several studies have demonstrated that *hly*III is widespread in lactobacilli, and that these microbes do not show any hemolytic activity [37]. Therefore, we concluded that *hly*III in PMO 08 is not a safety concern.

The second category includes genes that produce D-lactate, which may cause metabolic stress in patients [38]. In the KEGG database, two genes were identified as D-lactate producers in PMO 08 and their enzymes were D-lactate dehydrogenase and lactate racemase (Table 2). Recently, several studies have reported that both L- and D-lactate can be metabolized in the human body and are regarded as safe for healthy individuals [39]. However, Monroe (2019) [40] reported that D-lactate can cause acidosis in infants and patients with short bowel syndrome [40]. Therefore, a general precaution regarding D-lactate-producing bacteria is necessary for infants or patients with short bowel syndrome [41].

**Fig 4:**
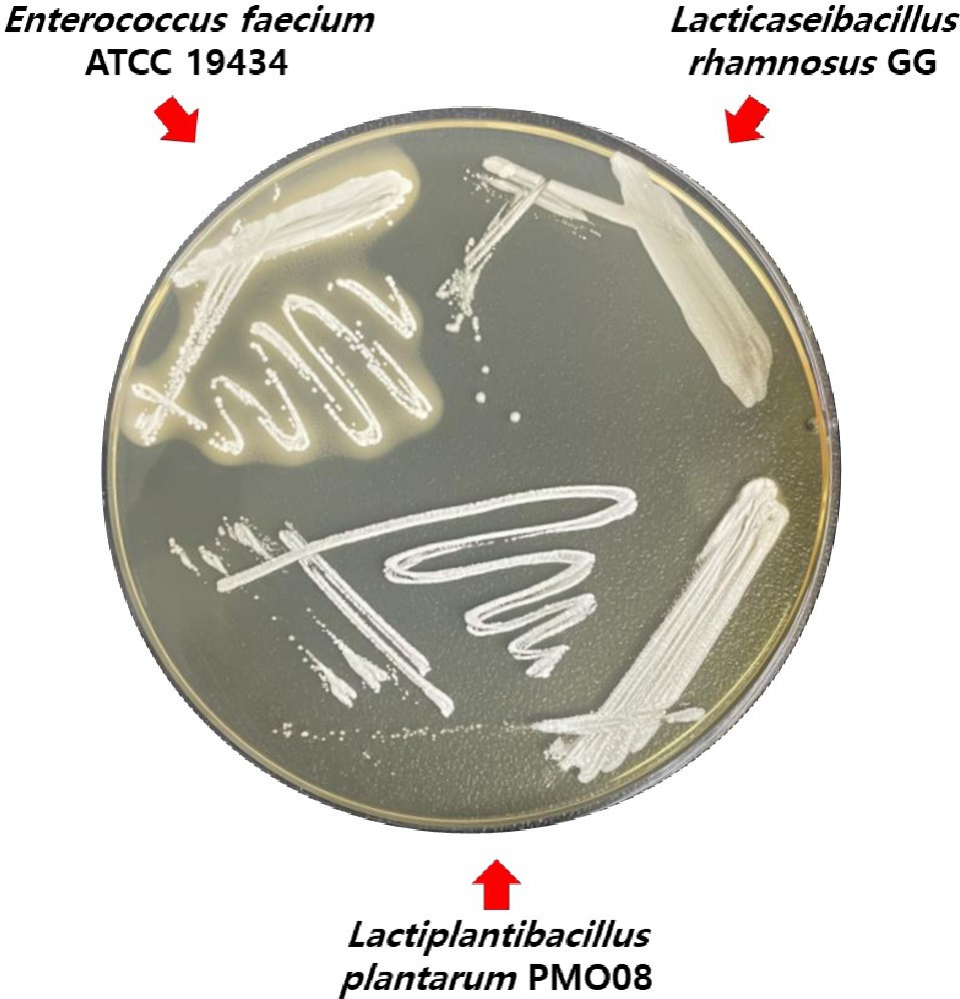
Bile salt deconjugation activity of *Lactiplantibacillus plantarum* PMO 08 on MRS medium containing 0.5% taurodeoxycholic acid. Plates were incubated anaerobically at 37°C for 48 h. *Enterococcus faecium* ATCC 19434 used as a positive control and *Lacticaseibacillus rhamnosus* GG used as a negative control.

The third category includes genes that produce secondary bile salts that can promote colorectal cancer [42]. Secondary bile salts, such as deoxycholic acid, can be synthesized by bile salt hydrolase (EC 3.5.1.24), which hydrolyzes the amide bond of 7α-dehydroxylated primary bile salt [43]. In the KEGG database, three choloylglycine hydrolases (bile salt hydrolases) were identified as being involved in primary or secondary bile acid biosynthesis in the PMO 08 strain. However, when the bile salt deconjugation activity of PMO 08 was tested on MRS agar plates containing 0.5% taurodeoxycholic acid, the strain showed no activity (Fig 4). These results indicated that they do not contribute to the synthesis of secondary bile salts. According to the guidelines for the evaluation of probiotics in food issued by the FAO/WHO, bile salt hydrolase is one of the desirable properties for probiotics because strains with this enzyme are resistant to gastric conditions [44]. Therefore, we concluded that bile salt hydrolase and its gene in PMO 08 are not a safety concern.

The last category includes genes that produce biogenic amine such as histamine through amino acid metabolism which can cause hypotension, flushing, and headache [45]. In the KEGG database, no biogenic producing genes were detected.

### 3.4 Antibiotic resistance

To analyze the antibiotic resistance of *Lp*. *plantarum* PMO 08, a MIC test was performed according to the EFSA guidelines (Table 3). Among the eight antibiotics tested, ampicillin exceeded the cutoff value, with a MIC of 4 μg/mL. Ampicillin belongs to the β-lactam antibiotic group and acts as an inhibitor of transpeptidase, which is essential for cell wall assembly [46].

**Table 3:** Antibiotic resistance of *Lactiplantibacillus plantarum* PMO 08.

Antibiotics	MIC^1^ (μg/mL)	EFSA cut off^2^ (μg/mL)
Ampicillin	4	2
Vancomycin	>128	NR
Gentamicin	2	16
Kanamycin	64	64
Streptomycin	32	NR
Erythromycin	0.5	1
Clindamycin	0.5	8
Tetracyclin	32	32
Chloramphenicol	8	8

^1^MIC: Minimum inhibitory concentration.

^2^Microbiological cut-off values for *Lp*. *plantarum* according to EFSA 2012. NR, not required.

The primary concern regarding antibiotic resistance genes in lactic acid bacteria is the possibility of transferring such genes to other bacteria via mobile elements, such as plasmids or bacteriophages. Therefore, when the MIC value is above the cut-off value, it is necessary to determine whether these genes are intrinsic or acquired resistance through genetic analysis.

The presence of antibiotic resistance genes was analyzed using BlastKoala, CARD database, and ResFinder 4.0, and their transfer possibilities were investigated by PHASTER database and Mobile Element Finder (Tables 4 and 5). In BlastKoala, 14 genes related to antibiotic resistance were detected in the chromosome, but not in the plasmids. In CARD and ResFinder, no antibiotic resistance genes were detected in criteria with strict or perfect (S1 Table). In phage analysis, six phage regions were detected: three in the chromosome, two in plasmid 1, and one in plasmid 2 (Table 5). In addition, in mobile element analysis, nine regions were determined: five ISP2, one ISP1, and three ISLhe30, and all of them were insertion sequence (Table 6). However, antibiotic resistance genes were not present in these phage regions or mobile element sequence indicating a low possibility of transfer to other bacteria.

**Table 4:** List of antimicrobial resistance genes analyzed by BlastKoala and their locations in the genome of *Lactiplantibacillus plantarum* PMO 08.

No.	Resistance	KEGG ID	Gene name	Protein ID	Location
1	Tetracycline resistance	K18220	ribosomal protection tetracycline resistance protein	QOF03178.1	Chromosome
2	Macrolide resistance	K18231	macrolide transport system ATP-binding/permease protein	QOF03071.1	
3	Phenicol resistance	K19271	chloramphenicol O-acetyltransferase type A	QOF01847.1	
4	Beta-Lactam resistance	K17836	beta-lactamase class A	QOF02877.1	
				QOF01418.1"	
5	Vancomycin resistance	K07260	zinc D-Ala-D-Ala carboxypeptidase	QOF02491.1	
6	Vancomycin resistance	K08641	zinc D-Ala-D-Ala dipeptidase	QOF02677.1	
7	CAMP resistance	K03367	D-alanine—poly (phosphoribitol) ligase subunit 1	QOF01670.1	
8	CAMP resistance	K03739	membrane protein involved in D-alanine export	QOF01671.1	
9	CAMP resistance	K14188	D-alanine—poly (phosphoribitol) ligase subunit 2	QOF02140.1	
				QOF01672.1	
10	CAMP resistance	K03740	D-alanine transfer protein	QOF01673.1	
11	CAMP resistance	K14205	phosphatidylglycerol lysyltransferase	QOF02751.1	
12	Multidrug resistance	K18907	GntR family transcriptional regulator	QOF02139.1	
13	Multidrug resistance	K18104	ATP-binding cassette, subfamily B, bacterial AbcA/BmrA	QOF00967.1	
				QOF01313.1	
14	Multidrug resistance	K18908	multidrug efflux pump	QOF03170.1	

**Table 5:** List of prophage regions in the genome of *Lactiplantibacillus plantarum* PMO 08 predicted by PHASTER.

Region		Region length	Completeness	Score	Total proteins	Region position	Most common phage	GC %
Chromosome	1	50.9 kb	intact	130	53	366,380–417,318	PHAGE_Lister_B025_NC_009812	42.16
	2	52.9 kb	intact	120	63	2,674,731–2,727,668	PHAGE_Oenoco_phiS13_NC_023560	41.41
	3	8.4 kb	incomplete	50	6	2,930,277–2,938,775	PHAGE_Entero_4MG_NC_022968	44.26
Plasmid 1	1	8.8 kb	incomplete	30	8	1,466–10,302	PHAGE_Erwini_pEp_SNUABM_01_NC_048807	40.51
	2	7.7 kb	incomplete	40	8	35,674–43,378	PHAGE_Bacill_G_NC_023719	40.91
Plasmid 2	1	15.7Kb	incomplete	40	12	3,464–19,250	PHAGE_Lactob_Lpa804_NC_048134	42.27

**Table 6:** List of mobile element regions in the genome of *Lactiplantibacillus plantarum* PMO 08 predicted by mobile genetic finder.

No.	Name	Type	Allele_len	e_value	Identity	Coverage	Region position
1	ISP2	Insertion sequence	1794	0	0.98	1.00	1,706,847–1,708,640
2	ISP2	Insertion sequence	1794	0	0.99	1.00	316,189–317,982
3	ISP2	Insertion sequence	1794	0	0.99	1.00	2,933,960–2,935,753
4	ISP2	Insertion sequence	1796	0	0.99	1.00	1,934,456–1,936,251
5	ISP2	Insertion sequence	1796	0	0.99	1.00	1,609,319–1,611,114
6	ISP1	Insertion sequence	1433	0	0.99	1.00	2,937,620–2,939,052
7	ISLhe30	Insertion sequence	1010	0	0.95	0.98	899,753–900,762
8	ISLhe30	Insertion sequence	1010	0	0.95	0.98	1,119,428–1,120,437
9	ISLhe30	Insertion sequence	1010	0	0.95	0.98	1,271,053–1,272,062

Intrinsic antibiotic resistance is chromosomally encoded and not transferable to other bacteria [47]. In case of vancomycin, the resistance or sensitivity is from intrinsic nature of bacteria such as *Enterococcus*, *Lactobacillus*, and *Bifidobacterium*; their peptidoglycan cell walls have D-Ala-D-Lac termini that have low binding affinity to vancomycin [48, 49].

In contrast, acquired antibiotic resistance located on mobile genetic elements, such as plasmids or phage regions, can be transferred from one bacterium to another, contributing to a greater and more widespread risk than intrinsic resistance [47]. When we analyzed the presence of antibiotic resistance genes in the genome of the PMO 08 strain, we found that they were located on the chromosome, but not in the plasmid or phage regions. Therefore, we concluded that PMO 08 has a low possibility of transferring antibiotic-resistance genes to other bacteria.

### 3.5 Carbohydrate utilization pathway and genes

**Fig 5:**
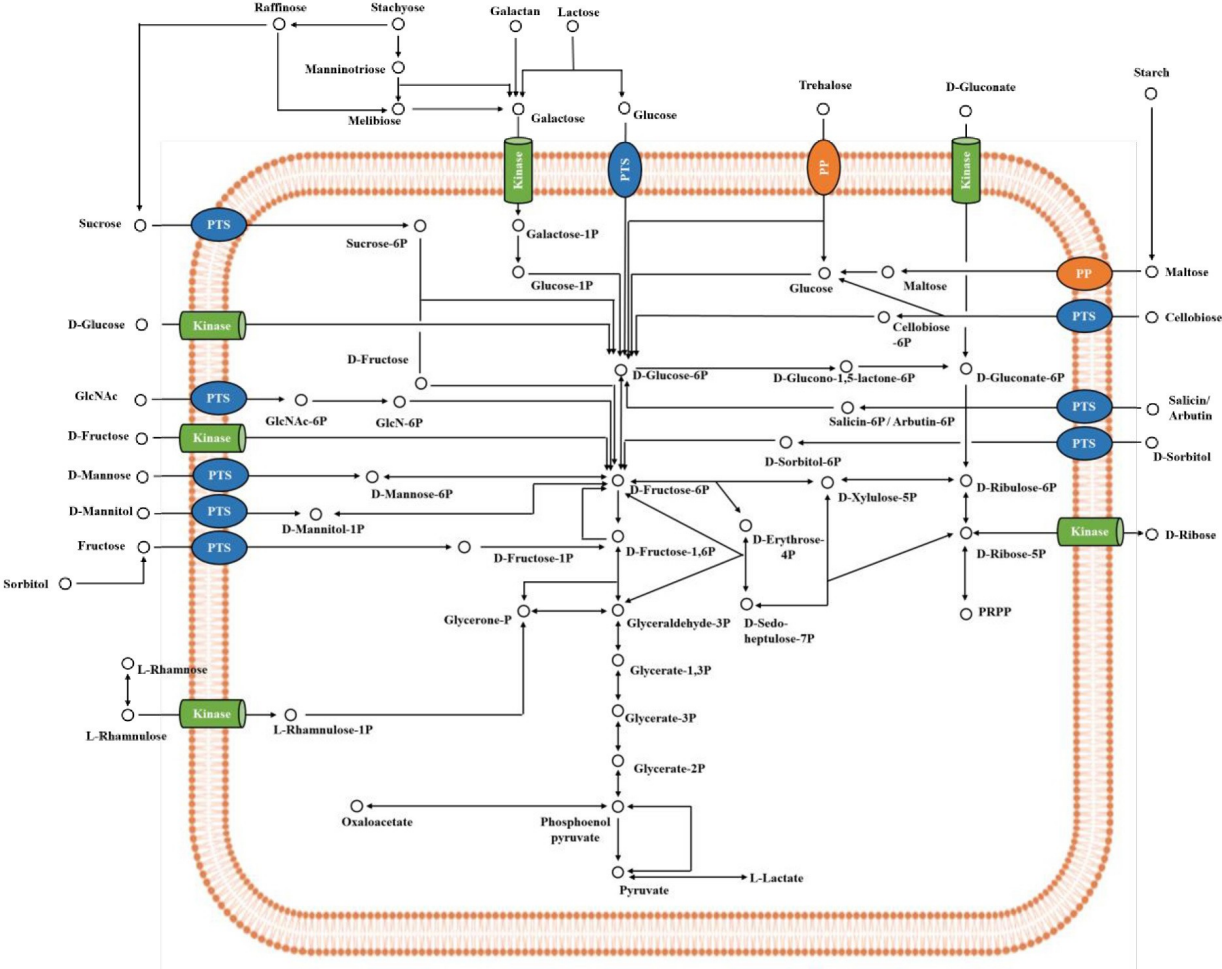
*In silico* carbohydrate utilization pathways of *Lactiplantibacillus plantarum* PMO 08 constructed by whole genome sequences. PTS, phosphotransferase; PP, phosphorylase.

To investigate the metabolic characteristics of *Lp*. *plantarum* PMO 08, carbohydrate utilization capacity was analyzed using 49 substrates (Table 6) and matched at the genome level using KEGG pathway analysis (Fig 5 and S2 Table). PMO 08 and WCFS1 commonly utilized L-arabinose, D-ribose, D-galactose, D-glucose, D-fructose, D-mannose, mannitol, sorbitol, N-acetyl-glucosamine, amygdalin, arbutin, esculin, salicin, cellobiose, maltose, lactose, melibiose, sucrose, trehalose, melizitose, raffinose, gentibiose, turanose, and tagatose. Notably, PMO 08 could utilize both α-methyl-D-mannoside and gluconate, whereas WCFS1 could not. Accordingly, this result indicates that PMO 08 has different biochemical attributes than WCFS1 in terms of carbohydrate utilization patterns. In the glycolysis/gluconeogenesis pathway, PMO 08 possesses a PTS system and glucokinase to utilize glucose. In addition, this strain possesses a fructose PTS system, fructose kinase, mannose PTS system, and galactose kinase for sugar intake. These results match the carbohydrate utilization patterns seen in Table 7.

**Table 7:** Carbohydrate utilization profiles of *Lactiplantibacillus plantarum* PMO 08 analyzed by *in vitro* and *in silico* methods.

No	Carbohydrates	WCFS1	PMO 08	Metabolic pathway	No	Carbohydrates	WCFS1	PMO 08	Metabolic pathway
1	Glycerol	**-**	**-**	**?**	26	Salicin	**+**	**+**	**+**
2	Erythritol	**-**	**-**	**?**	27	Cellobiose	**+**	**+**	**+**
3	D-Arabinose	**-**	**-**	**+**	27	Maltose	**+**	**+**	**+**
4	L-Arabinose	**+**	**+**	-*	29	Lactose	**+**	**+**	**+**
5	Ribose	**+**	**+**	**+**	30	Melibiose	**+**	**+**	**+**
6	D-Xylose	**-**	**-**	**-**	31	Sucrose	**+**	**+**	**+**
7	L-Xylose	**-**	**-**	**-**	32	Trehalose	**+**	**+**	**+**
8	Ribitol	**-**	**-**	**-**	33	Inulin	**-**	**-**	**-**
9	β-Methyl-xylose	**-**	**-**	**?**	34	Melezitose	**+**	**+**	**?**
10	Galactose	**+**	**+**	**+**	35	D-Raffinose	**+**	**+**	**+**
11	D-Glucose	**+**	**+**	**+**	36	Starch	**-**	**-**	+*
12	D-Fructose	**+**	**+**	**+**	37	Glycogen	**-**	**-**	+*
13	D-Mannose	**+**	**+**	**+**	38	Xylitol	**-**	**-**	**-**
14	L-Sorbose	**-**	**-**	**-**	39	Gentiobiose	**+**	**+**	**?**
15	Rhamnose	**-**	**-**	+*	40	D-Turanose	**+**	**+**	**?**
16	Galactitol	**+**	**+**	-*	41	D-Lyxose	**-**	**-**	**-**
17	Inositol	**-**	**-**	**-**	42	D-Tagatose	**+**	**+**	-*
18	Mannitol	**+**	**+**	**+**	43	D-Fucose	**-**	**-**	**-**
19	Sorbitol	**+**	**+**	**+**	44	L-Fucose	**-**	**-**	**-**
20	α-Methyl-D-mannoside	**-**	**+**	**?**	45	D-Arabitol	**-**	**-**	**-**
21	α-Methyl-D-glucoside	**-**	**-**	**-**	46	L-Arabitol	**-**	**-**	**-**
22	N-Acetyl-glucosamine	**+**	**+**	**+**	47	Gluconate	**△**	**+**	**+**
23	Amygdalin	**+**	**+**	**+**	48	2-Keto-gluconate	**-**	**-**	**?**
24	Arbutin	**+**	**+**	**+**	49	5-Keto-gluconate	**-**	**-**	**?**
25	Esculin	**+**	**+**	**+**					

*In vitro* carbohydrate utilization of PMO 08 and WCFS1 was examined using an API CHL kit. The results are presented according to the color chart provided by the manufacturer: -, negative; △, weakly positive; +, positive. Metabolic pathways of PMO 08 for each carbohydrate were matched at the genome level using the KEGG database: -, incomplete; +, complete; ?, no data in KEGG;

*, *in silico* results are not matched with those of *in vitro* analysis

However, PMO 08 could not consume L-rhamnose, even though this strain shows a complete pathway to utilize this deoxy sugar. Moreover, this strain has an incomplete pathway to utilize tagatose, but can metabolize that carbon source. In the pentose phosphate pathway, PMO 08 harbors ribokinase to metabolize ribose. However, it does not carry the complete pathway to consume the D/L- form of arabinose or xylose, and these results correspond to those in Table 7, except for L-arabinose. PMO 08 has an incomplete L-arabinose pathway but can utilize that carbon source *in vitro*. For disaccharide and oligosaccharide metabolism, PMO 08 uses a sucrose PTS system, cellobiose PTS system, maltose phosphorylase, and trehalose phosphorylase to utilize these carbon sources. In addition, this strain possesses α- or β-galactosidase to utilize lactose, melibiose, and raffinose. These results correspond with those of the *in vitro* tests shown in Table 7.

However, in the case of polysaccharides, PMO 08 has a complete pathway for utilizing starch and glycogen, but the strain cannot metabolize these polysaccharides. In addition, this strain does not carry the genes to consume inulin, and this result is consistent with the experimental data in Table 7. In the case of sugar alcohols, PMO 08 has a mannitol and sorbitol PTS system to utilize these carbon sources. However, it possesses an incomplete pathway to use ribitol, galactitol, inositol, xylitol, and D/L- form of arabitol, and these results were matched with the *in vitro* test in Table 7, except for galactitol. Although the strain can metabolize galactitol in the experiment, the related genes were not found in the KEGG analysis. In amino sugar and nucleotide sugar metabolism, PMO 08 has a sugar PTS system to metabolize N-acetylglucosamine, which corresponds with the *in vitro* test. Lastly, PMO 08 carries several β-glucosidase enzymes that use glucosides such as amygdalin, arbutin, esculin, and salicin, which is consistent with the results shown in Table 7. PMO 08 could utilize α-methyl-D-mannoside, whereas WCFS1 could not. Considering the origin of PMO 08 (kimchi, [10]), which is rich in hemicellulose, compared to that of WCFS1 (human saliva, [50]). PMO 08 has multiple copies of mannose PTS system EII components while WCFS1 has one copy of that gene (S2 Table). In addition, the PMO 08 harbors an inositol monophosphatase family protein, which is related with phytate hydrolysis [51]. Taken together, the experimental results were consistent with genome analysis in many carbon sources, but some (L-arabinose, rhamnose, galactitol, starch, glycogen, and D-tagatose) were not matched; thus, further studies are required.

### 3.6 Probiotic characteristics

To verify the probiotic characteristics of *Lp*. *plantarum* PMO 08 at the genomic level, genes related to probiotic properties were annotated using NCBI Prokaryotic Genome Annotation Pipeline (PGAP v4.11). As shown in Table 8, several genes related to acid or bile salt tolerance, adhesion to epithelial cells, and stress scavenging were found on the chromosome of PMO 08.

**Table 8:** Genes related with probiotic characteristics in genome of *Lactiplantibacillus plantarum* PMO 08.

Function	Locus tag	Protein ID	Annotation
Acid tolerance	IGB08_04050	QOF02569.1	monovalent cation:proton antiporter family protein
	IGB08_04420	QOF02637.1	sodium:proton antiporter
	IGB08_06005	QOF02922.1	cation:proton antiporter
	IGB08_06875	QOF03090.1	Na+/H+ antiporter NhaC
	IGB08_09030	QOF00692.1	Na+/H+ antiporter NhaC
	IGB08_09865	QOF00846.1	cation:proton antiporter
	IGB08_11065	QOF01073.1	cation:proton antiporter
	IGB08_11410	QOF01136.1	sodium:proton antiporter
	IGB08_11700	QOF01193.1	sodium:proton antiporter
	IGB08_11930	QOF01238.1	Na+/H+ antiporter
	IGB08_12820	QOF01395.1	F0F1 ATP synthase subunit A
	IGB08_12825	QOF01396.1	F0F1 ATP synthase subunit C
	IGB08_12830	QOF01397.1	F0F1 ATP synthase subunit B
	IGB08_12835	QOF01398.1	F0F1 ATP synthase subunit delta
	IGB08_12840	QOF01399.1	F0F1 ATP synthase subunit alpha
	IGB08_12845	QOF01400.1	F0F1 ATP synthase subunit gamma
	IGB08_12850	QOF01401.1	F0F1 ATP synthase subunit beta
	IGB08_12855	QOF01402.1	F0F1 ATP synthase subunit epsilon
	IGB08_04820	QOF02713.1	chaperonin GroEL
	IGB08_04825	QOF02714.1	co-chaperone GroES
Bile salt tolerance	IGB08_07390	QOF03186.1	choloylglycine hydrolase family protein
	IGB08_08270	QOF00553.1	choloylglycine hydrolase family protein
	IGB08_08930	QOF00673.1	choloylglycine hydrolase family protein
	IGB08_12030	QOF01257.1	linear amide C-N hydrolase
	IGB08_00055	QOF01840.1	GNAT family N-acetyltransferase
	IGB08_00185	QOF01864.1	GNAT family N-acetyltransferase
Adhesion	IGB08_13995		elongation factor Tu
	IGB08_05625	QOF02860.1	class A sortase
	IGB08_01445	QOF02101.1	LPXTG cell wall anchor domain-containing protein
	IGB08_02835	QOF02342.1	tyrosine-protein phosphatase
	IGB08_02840	QOF02343.1	CpsD/CapB family tyrosine-protein kinase
	IGB08_07380	QOF03184.1	tyrosine-protein phosphatase
	IGB08_09285	QOF00736.1	tyrosine-protein phosphatase
	IGB08_11465	QOF01147.1	tyrosine-protein phosphatase
	IGB08_14050	QOF01601.1	CpsD/CapB family tyrosine-protein kinase
	IGB08_14055	QOF03340.1	tyrosine protein phosphatase
	IGB08_03205	QOF02413.1	flagellar biosynthesis protein FlhB
Stress	IGB08_13050	QOF01437.1	thiol peroxidase
	IGB08_06755	QOF03067.1	glutathione peroxidase
	IGB08_12965	QOF01421.1	glutamate—cysteine ligase GshA/glutathione synthetase GshB
	IGB08_07420	QOF03192.1	arsenate reductase (thioredoxin)
	IGB08_08685	QOF00631.1	thioredoxin family protein
	IGB08_11850	QOF01222.1	thioredoxin family protein
	IGB08_13250	QOF01475.1	thioredoxin
	IGB08_04655	QOF02683.1	thioredoxin-disulfide reductase
	IGB08_06680	QOF03052.1	thioredoxin
	IGB08_04525	QOF02658.1	triose-phosphate isomerase

Na+/H+ antiporters, cation:proton antiporters, F0F1 ATP synthase subunits, and chaperonin GroEL were annotated as contributing resistance to low pH in PMO 08. These genes are associated with the regulation of cytoplasmic pH by hydrolyzing ATP to pump H+ from cells, which allows for the maintenance of pH homeostasis [52]. Generally, F0F1 ATP synthase subunits are highly expressed under low pH conditions [53]. Choloylglycine hydrolase (bile salt hydrolase) and GCN5-*N*-acetyltransferase (GNAT) proteins were found to be related to bile salt tolerance in PMO 08. Bile salt hydrolase catalyzes the hydrolysis of bile salts and is thought to be involved in the bile salt resistance of bacteria [54]. In addition, studies have reported that bile salt hydrolase can lower cholesterol levels by facilitating the integration of cholesterol or bile into the bacterial membrane [55]. GNAT family proteins contribute to the maintenance of resistance to bile salts through acetylation of the outer membrane glycolipid enterobacterial common antigen [56].

Another essential probiotic characteristic is the ability to adhere to the epithelial membrane. In the genome analysis of PMO 08, elongation factor, LPXTG cell wall anchor domain-containing protein, tyrosine protein phosphatase, CpsD/CapB family tyrosine-protein kinase, and flagellar biosynthesis proteins were annotated as adhesion-related proteins. Among them, the elongation factor Tu acts as an aminoacyl tRNA binding to the active site of ribosomes and this indicates the adhesive ability of PMO 08 to mucin [57]. LPXTG cell wall anchor domain-containing proteins or tyrosine-protein phosphatases act as mucus adhesins to mediate binding to human epithelial cells or adhesive signaling functions [58, 59]. Finally, several antioxidant-related genes, such as triose-phosphate isomerase, thiol peroxidase, glutathione peroxidase, and thioredoxin family proteins were detected in the PMO 08 strain. In case of triose-phosphate isomerase, it functions as an antioxidant via glutathionylation [60]. Furthermore, the thioredoxin-thioredoxin reductase and GSH- glutaredoxin systems, which maintain intracellular dithiol/disulfide homeostasis in lactic acid bacteria, play an important role in the defense against oxidative stress. The thioredoxin system, comprising NADPH, thioredoxin reductase, and thioredoxin, shuttles electrons to thiol-dependent peroxidases to maintain redox homeostasis and protect probiotic bacteria from ROS and RNS damage [61]. This system controls the thiol-disulfide balance and thus plays an essential role in DNA and protein repair by reducing ribonucleotide reductase, methionine sulfoxide reductases, and regulating the activity of numerous redox-sensitive transcription factors [62]. Overall, these results suggest that *Lp*. *plantarum* PMO 08 has probiotic characteristics and defense mechanism against oxidative stress that can be verified using *in silico* genomic analysis.

## 4. Conclusion

*Lp*. *plantarum* PMO 08 has been used as a plant-based probiotic starter, but further investigation is limited because of the absence of information regarding whole genome sequences. In this study, we analyzed the whole genome sequence of the PMO 08 strain and found that it contains one chromosome and two plasmids with a high homology (≥ 99%) of ANI value to *Lp*. *plantarum* WCFS1. We conducted safety analysis both *in vitro* and *in silico*, and no deleterious genes were detected. In the case of antibiotic resistance genes, PMO 08 was resistant to ampicillin, but these genes were not transferable. In addition, PMO 08 can utilize various carbohydrates that are favorable for adaptation to the plant environment, and these results are consistent with carbohydrate utilization enzymes at the genomic level. The strain also harbors a gene for inositol monophosphatase family protein related with phytate hydrolysis. Furthermore, we verified probiotic characteristics, such as acid or bile salt tolerance, adhesion to epithelial cells, and stress scavenging of PMO 08 at the genomic level. This study demonstrates that *Lp*. *plantarum* PMO 08 contains several probiotic-related genes (while harboring no deleterious genes) and is a suitable probiotic starter for plant-based fermentation. This information will provide considerable insight into the physiology of this organism and make it possible to understand their genetic content, predict important capabilities, and manipulate it for improved beneficial activities.

## Supporting information

S1 Fig COG analysis of annotated genes for *Lactiplantibacillus plantarum* PMO 08. Functional categories were presented on the right part of the figure with blanket which shows number of genes and percentage.
(DOCX)

S1 Table Antimicrobial resistance genes determined by CARD database and ResFinder sever 4.1. (XLSX)

S2 Table Comparison of enzyme related with carbohydrate utilization between *Lactiplantibacillus plantarum* PMO 08 and *Lp*. *plantarum* WCFS1. (XLSX)
