# Supplementary material for: Integrated genome-based assessment of safety and probiotic characteristics of Lactiplantibacillus plantarum PMO 08 isolated from kimchi
Source: PLoS One. 2022 Oct 3;17(10):e0273986. doi: 10.1371/journal.pone.0273986 (PMC9529155; doi:10.1371/journal.pone.0273986)
Supplement: S1 Fig — Functional categories were presented on the right part of the figure with blanket which shows number of genes and percentage. (DOCX) [file pone.0273986.s001.docx]

**
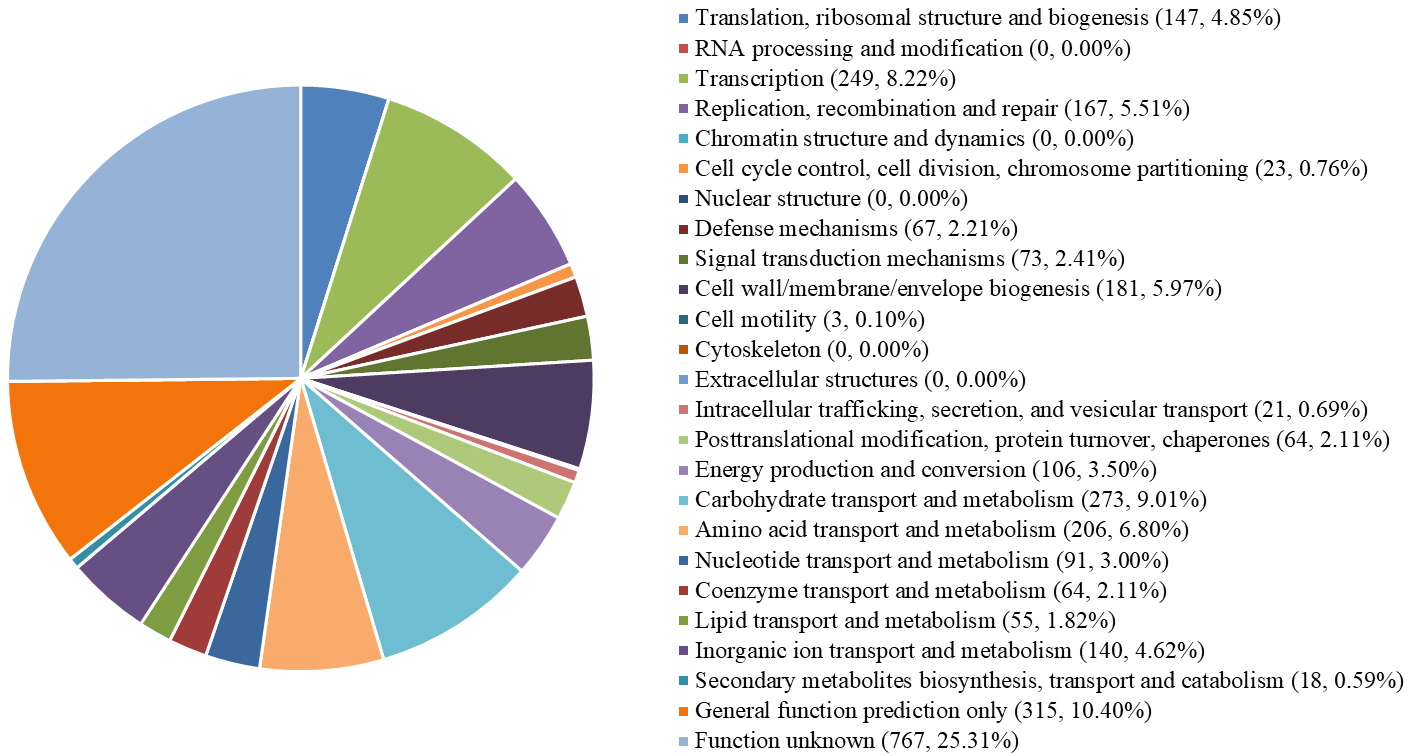
**

**S1 Fig. COG analysis of annotated genes for *Lactiplantibacillus plantarum* PMO 08. Functional categories were presented on the right part of the figure with blanket which shows number of genes and percentage.**
